# Differential expression of copper-associated and oxidative stress related proteins in a new variant of copper toxicosis in Doberman pinschers

**DOI:** 10.1186/1476-5926-4-3

**Published:** 2005-03-24

**Authors:** Bart Spee, Paul JJ Mandigers, Brigitte Arends, Peter Bode, Ted SGAM van den Ingh, Gaby Hoffmann, Jan Rothuizen, Louis C Penning

**Affiliations:** 1 Department of Clinical Sciences of Companion Animals, Faculty of Veterinary Medicine, Utrecht University, The Netherlands; 2 Interfacultary Reactor Institute, Delft University, The Netherlands; 3 Department of Pathobiology, Faculty of Veterinary Medicine, Utrecht University, The Netherlands

## Abstract

**Background:**

The role of copper accumulation in the onset of hepatitis is still unclear. Therefore, we investigated a spontaneous disease model of primary copper-toxicosis in Doberman pinschers so to gain insights into the pathophysiology of copper toxicosis, namely on genes involved in copper metabolism and reactive oxygen species (ROS) defences.

**Results:**

We used quantitative real-time PCR to determine differentially expressed genes within a target panel, investigating different groups ranging from copper-associated subclinical hepatitis (CASH) to a clinical chronic hepatitis with high hepatic copper concentrations (Doberman hepatitis, DH). Furthermore, a non-copper associated subclinical hepatitis group (N-CASH) with normal hepatic copper concentrations was added as a control. Most mRNA levels of proteins involved in copper binding, transport, and excretion were around control values in the N-CASH and CASH group. In contrast, many of these (including ATP7A, ATP7B, ceruloplasmin, and metallothionein) were significantly reduced in the DH group. Measurements on defences against oxidative stress showed a decrease in gene-expression of superoxide dismutase 1 and catalase in both groups with high copper. Moreover, the anti-oxidative glutathione molecule was clearly reduced in the DH group.

**Conclusion:**

In the DH group the expression of gene products involved in copper efflux was significantly reduced, which might explain the high hepatic copper levels in this disease. ROS defences were most likely impaired in the CASH and DH group. Overall, this study describes a new variant of primary copper toxicosis and could provide a molecular basis for equating future treatments in dog and in man.

## Background

Copper is an imperative molecule in life; in contradiction, however, it is highly toxic [1]. Like zinc, iron, and selenium, copper is an essential trace element in diets and is required for the activity of a number of physiologically important enzymes [2]. Cells have highly specialized and complex systems for maintaining intracellular copper concentrations [3]. If this balance is disturbed, excess copper can induce oxidative stress that could lead to chronic inflammation [4,5]. Copper induced hepatitis has been described both in humans (Wilson's disease) as well as in dogs. There are several non-human models of copper toxicosis models, such as the Long-Evans Cinnamon rats and Bedlington terriers. Although the gene underlying Wilson's disease (ATP7B) is deficient in Long-Evans Cinnamon rats [6-9], in Bedlington terriers it has been excluded as a candidate for copper toxicosis [10]. The recent discovery of mutations in gene MURR1, responsible for copper toxicosis in Bedlington terriers, has given rise to the discovery of a new copper pathway [11]. Here, we describe in Doberman pinschers a copper associated chronic hepatitis (also called Doberman hepatitis), characterized by micro-nodular cirrhosis with elevated hepatic copper concentrations [12-15]. Doberman hepatitis accounts for 4 % of all deaths in a Dutch population of 340 Dobermans [16]. Until recently, the role of copper in the development and progression of hepatitis in the Doberman pinscher had been unclear. Recent studies using intravenous ^64^Cu clearly show an impaired copper excretion in dogs with hepatitis and elevated copper concentrations [17]. However, genes ATP7B and MURR1 have been excluded by us as possible candidates by genotyping (data not shown). Therefore, Doberman hepatitis can be seen as a separate form of copper toxicosis and a possible model for other types of copper toxicosis in humans, such as Indian childhood cirrhosis, non-Indian childhood cirrhosis, or idiopathic copper toxicosis.

**Figure 1:**
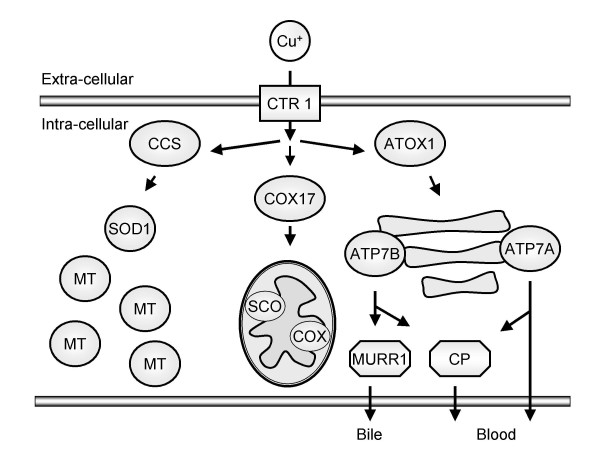
**Schematic overview of intra-cellular copper trafficking in hepatocytes. **Copper uptake is mediated by the receptor CTR1. In the cell, copper can bind to copper chaperones such as CCS, COX17, and ATOX1 which in turn deploy to SOD1, the mitochondrial COX, and ATP7A/B, respectively. ATP7A can directly excrete copper or bind it to ceruloplasmin (CP). ATP7B can excrete copper through CP to blood or via MURR1 to bile. Furthermore, metallothioneins (MT) are present in the cytoplasm which can bind and sequester metals. [SCO are metallochaperone proteins with essential, but not yet fully understood, roles in copper delivery to mitochondrial COX.]

Intracellular copper is always transiently associated with small copper-binding proteins (Figure 1), denoted copper chaperones, which distribute copper to specific intracellular destinations [18]. One of these copper chaperones is the anti-oxidant protein 1 (ATOX1) [19], which transports copper to the copper-transporting ATPases ATP7A and ATP7B [20], located in the trans-Golgi network. Copper can then be bound to liver specific ceruloplasmin (CP) [21] or MURR1 and transferred outside the cell to blood and bile, respectively [22]. The second chaperone – cytochrome c oxidase (COX17) is responsible for delivering copper to the mitochondria for incorporation into cytochrome c oxidase [23]. The third chaperone – copper chaperone for superoxide oxidase (CCS) is responsible for the incorporation of copper into Cu/Zn superoxide dismutase (SOD1) – one of the most important cytosolic enzymes in the defence against oxidative stress [24,25]. Also known as ferroxidase or oxygen oxidoreductase, CP is a plasma metalloprotein which is involved in peroxidation of Fe(II)transferrin to Fe(III)transferrin and forms 90 to 95 % of plasma copper. CP is synthesized in hepatocytes and is secreted into the serum with copper incorporated during biosynthesis. Metallothionein 1A (MT1A) is a small intracellular protein capable of chelating several metal ions, including copper. It contains many cysteine residues, which allow binding and storage of copper. Furthermore, MT1A is inducible, at the transcriptional level, by metals and a variety of stressors such as reactive oxygen species (ROS), hypoxia, and UV radiation [26]. MT1A can donate copper to other proteins, either following degradation in lysosomes or by exchange via glutathione (GSH) complexation [27].

High hepatic levels of copper induce oxidative stress. There are several important proteins and molecules involved in the defence against oxidative stress. Most of the anti-oxidants can be grouped into either enzymatic defences or non-enzymatic defences [28]. The enzymatic defence against oxidative stress consists of several proteins that have tight regulations such as SOD1 and catalase (CAT). Non-enzymatic defences against oxidative stress consist of molecules such as α-tocopherol, β-carotene, ascorbate, and a ubiquitous low molecular thiol component – the GSH [29]. The present study was undertaken to investigate the effect of copper toxicosis on expression of gene-products involved in copper metabolism and oxidative stress in several gradations of hepatic copper toxicosis in Doberman pinschers.

## Results

To gain insight into the pathogenesis of copper toxicosis, we first measured mRNA levels on several important copper binding gene-products by means of quantitative real-time PCR (Q-PCR). Because copper toxicity is often associated with oxidative stress, we also measured several oxidative stress related gene-products. To determine a possible damaging effect of the oxidative stress, we investigated proteins involved in apoptosis and cell-proliferation.

### Gene-expression measurements on copper metabolism related gene products

**Figure 2:**
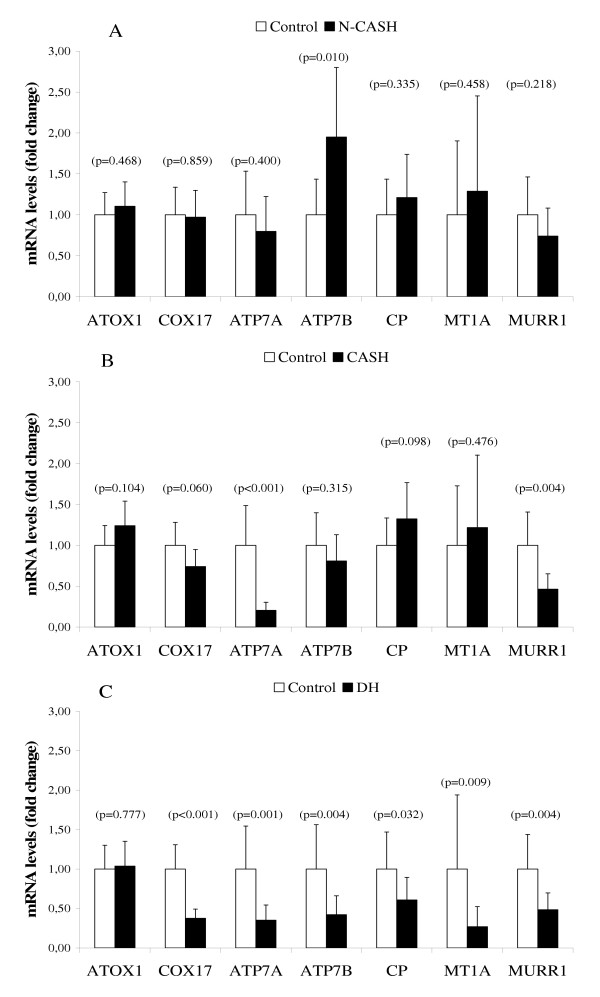
**Quantitative Real-Time PCR of copper metabolism related genes. **mRNA levels of non-copper associated subclinical hepatitis (n = 6 dogs) is shown in (A). mRNA levels of copper associated subclinical hepatitis (n = 6 dogs) is shown in (B). mRNA levels of Doberman hepatitis (n = 6 dogs) is shown in (C). Data represent mean ± 2 SD.

Several proteins in the Doberman hepatitis (DH) group are reduced compared to healthy controls (Figure 2C). In all groups the copper chaperone ATOX1 is not affected, whereas COX17 is decreased three-fold in the DH group and remains unchanged in the non-copper associated subclinical hepatitis group (N-CASH, Figure 2A) and copper associated subclinical hepatitis group (CASH, Figure 2B). In the DH group, the mRNA levels of both trans-Golgi copper transporting proteins ATP7A and ATP7B are decreased, three- and two-fold respectively. Interestingly, mRNA levels of ATP7A are decreased in the CASH group as well (Figure 2B). In contrast, ATP7B is not affected in the CASH group but is induced two-fold in the N-CASH group. CP mRNA levels are normal except for the DH group where it is decreased two-fold. The same observation was made with measurements on MT1A mRNA, although this protein is decreased four-fold in the DH group. The protein MURR1 (that transports copper from hepatocytes into bile) is unaffected in the N-CASH group but halved in the CASH and DH groups.

### Gene expression measurements on oxidative stress markers

**Figure 3:**
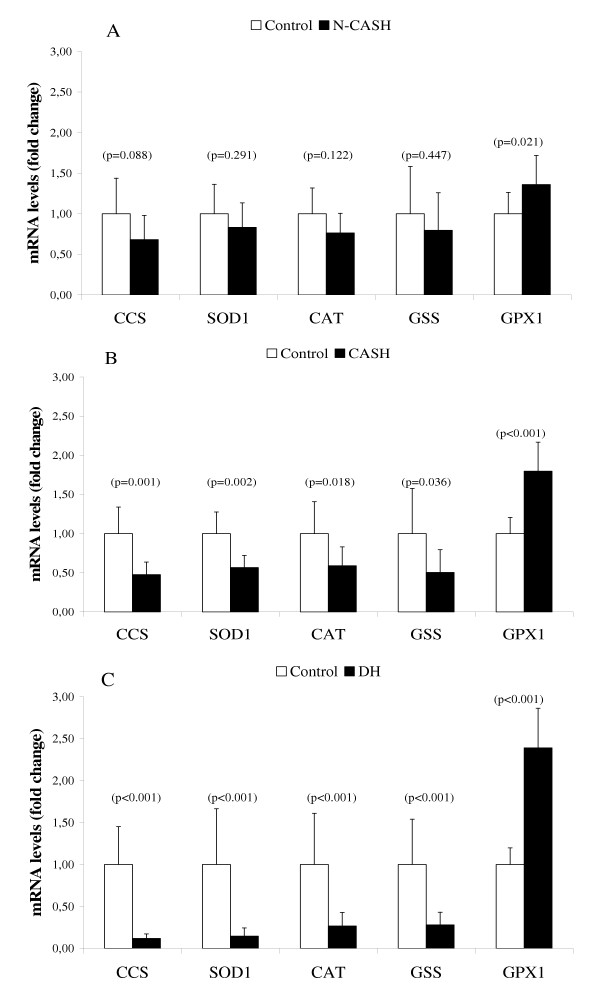
**Quantitative Real-Time PCR of oxidative stress markers. **mRNA levels of non-copper associated subclinical hepatitis (n = 6 dogs) is shown in (A). mRNA levels of copper associated subclinical hepatitis (n = 6 dogs) is shown in (B). mRNA levels of Doberman hepatitis (n = 6 dogs) is shown in (C). Data represent mean ± 2 SD.

SOD1 and CAT are reduced 7- and 4-fold (respectively) in the DH group when compared to healthy controls (Figure 3C). This reduction in mRNA levels can be seen in the CASH group (Figure 3B), where SOD1 and CAT are halved, but are not lowered significantly in the N-CASH group (Figure 3A). One of the GSH synthesis enzymes – the glutathione synthetase (GSS) is unaffected in the N-CASH group but reduced 2 to 4-fold in the CASH and DH group, respectively. The glutathione peroxidase (GPX1) responsible for converting oxidized glutathione (GSSG) into its reduced form (GSH) is induced slightly in mRNA expression in the N-CASH group, and is doubled in the CASH and DH groups. The third copper chaperone CCS, responsible for the transport of copper to SOD1, is inhibited 8-fold in the DH group, 2-fold in the CASH group, and remained unchanged in the N-CASH group.

### Gene expression measurements on apoptosis and cell proliferation

**Figure 4:**
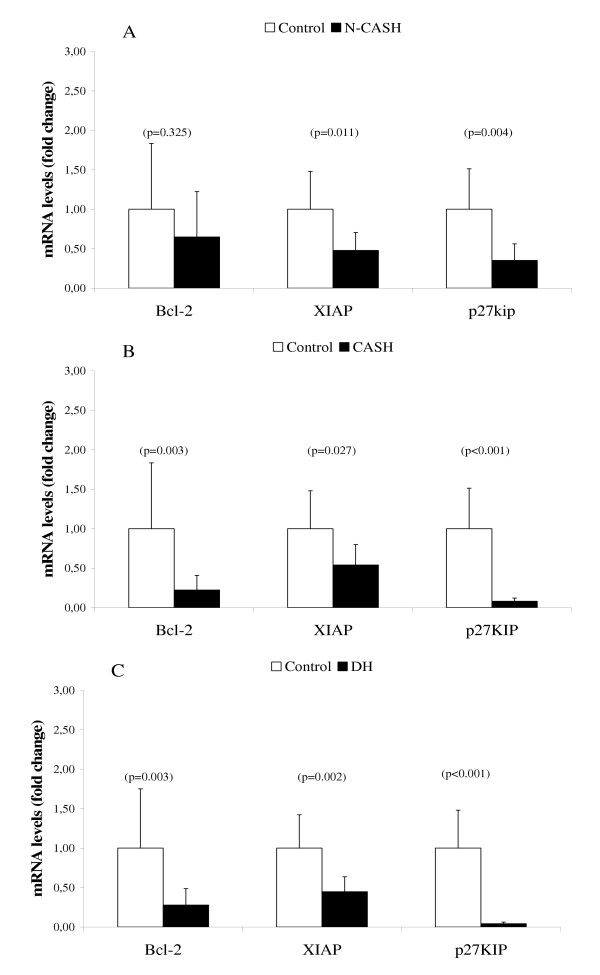
**Quantitative Real-Time PCR of apoptosis and cell proliferation related genes. **mRNA levels of non-copper associated subclinical hepatitis (n = 6 dogs) is shown in (A). mRNA levels of copper associated subclinical hepatitis (n = 6 dogs) is shown in (B). mRNA levels of Doberman hepatitis (n = 6 dogs) is shown in (C). Data represent mean ± 2 SD.

We measured two anti-apoptotic gene products, viz. Bcl-2, the frequently described anti-apoptotic protein, and a x-linked inhibitor of apoptosis (XIAP) recently associated with MURR1 [30]. Our apoptosis measurements on Bcl-2 showed no reduction in gene expression in the N-CASH group (Figure 4A), but is inhibited 4-fold in the CASH and DH groups (Figures 4B and 4C, respectively). XIAP is halved in all groups. The most dramatic changes were found in the mRNA levels of the cell-cycle inhibitor p27KIP which is inhibited 24-fold in the DH group, 12-fold in the CASH group, and 3-fold in the N-CASH group.

### Western blots analysis on metallothionein proteins during copper toxicosis

**Figure 5:**
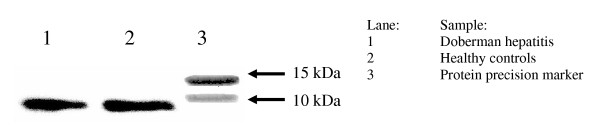
**Western blot analysis of the metallothionein proteins. **Immunoreactive bands of total metallothionein of pooled fractions of the Doberman hepatitis (DH) group (n = 6 dogs) *versus *healthy controls (n = 8 dogs).

Measurements on the mRNA levels of MT1A showed a marked decrease in gene expression in the DH group. In order to see whether this decrease was also occurring at the protein level, Western blots were performed in order to confirm decreased mRNA levels. Therefore, the total amount of metallothionein was determined from Doberman pinschers with chronic hepatitis and high copper (DH-group) levels compared to healthy Dobermans. Metallothionein was detected in both samples, where it was present as a single band of 6 kDa (Figure 5). Interestingly, the immunoreactive band shows no difference in concentration between the two samples.

### Total Glutathione measurements during copper toxicosis

**Figure 6:**
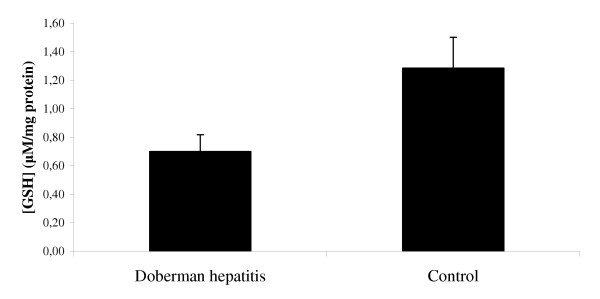
**Total glutathione (GSH) measurements during copper toxicosis in Doberman. **Total GSH levels of pooled protein fractions of the Doberman hepatitis (DH) group (n = 6 dogs) *versus *healthy controls (n = 8 dogs). Data represent mean ± 2 SD.

In order to determine whether the decrease in mRNA levels of GSS decreases the GSH levels, we measured the total amount of GSH. Interestingly, in Figure 6, the total amount of GSH in the high copper group is halved when compared to healthy controls.

## Discussion

In the present study, the expression of a total of 15 gene products involved in copper metabolism of Doberman pinschers was measured. This provided insight into the molecular pathways of a canine copper-associated hepatic disease model ranging from subclinical hepatitis with elevated copper levels (CASH) to severe chronic hepatitis with high hepatic copper levels (DH). Furthermore, these diseases were compared to non-copper associated subclinical hepatitis (N-CASH).

Because of the centrolobular accumulation of copper in the hepatocytes during copper toxicosis in the Doberman, a probable defect may be sought in the copper metabolism instead of a secondary effect due to, for instance, cholestasis. Recent findings by Mandigers *et al. *[17] indicated that Doberman pinschers with hepatitis and elevated copper concentrations suffer from impaired ^64^Cu bile excretion which is, together with other studies, conclusive that copper toxicosis exists in the Doberman pinscher. Furthermore, a double blind placebo-controlled study with the copper chelating agent, D-penicillamine, on Doberman pinschers with CASH showed a marked improvement of liver pathology [31]; currently, that agent is the only treatment option.

**Figure 7:**
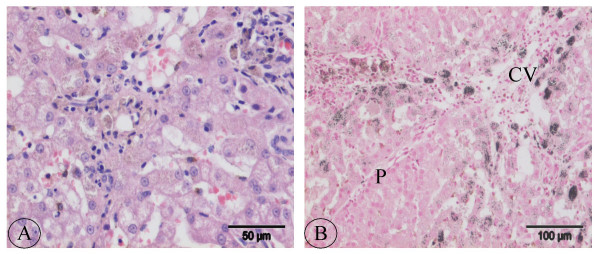
**Histological evaluation of Doberman hepatitis. **(A) Hepatitis characterised by accumulation of pigmented granules (probably copper) in hepatocytes, and inflammation with lymphocytes and pigmented (probably copper) macrophages. HE staining. (B) Centrolobular accumulation of copper in hepatocytes and band of fibrous tissue with inflammatory cells and copper-laden macrophages. Rubeanic acid staining. P = Portal area, CV = Central vein area.

If copper is sequestered, in time metallothioneins will store the copper in lysosomes, as described by Klein *et al*. [32]. They found that chronic copper toxicity in Long-Evans Cinnamon rats involved the uptake of copper-loaded metallothioneins into lysosomes, where it was incompletely degraded and polymerized into an insoluble material, which contained reactive copper. This copper initiated a lysosomal lipid peroxidation, which led to hepatocyte necrosis. Phagocytosis of this reactive copper by Kupffer cells amplified the liver damage. Histological examination of the DH (Figure 7) and CASH group samples revealed copper accumulation in hepatocytes and copper-laden Kupffer cells similar to that described by Klein *et al. *[32]; therefore, that can be denoted as benchmarks of chronic exposure to copper.

In our study, the gene expression levels of several gene products involved in copper metabolism seem to be reduced in the DH and CASH groups when compared to healthy controls. Short term studies on *in vitro *models all show an induction of MT1A or CP indicative of a higher efflux of copper from hepatocytes [33,34]. The reductions that are seen in our results could therefore be ascribed to the prolonged or chronic nature of copper accumulation as dogs in the high copper or DH group present clinical signs after 2 years. Therefore, our observations are not directly comparable with the short-term induced copper effects *in vitro*, but are clinically more relevant, showing the effects of long-term copper accumulation in Doberman hepatitis. However, Western blot experiments on metallothionein, which stores the copper in lysosomes, did not show any reduction at the protein level. This observation could be ascribed to the antibody that binds all metallothioneins, including metallothionein 2 (MT2A), which also is present in the liver. It remains to be proven if this effect is a compensation for the decrease of MT1A.

In the earlier stages of copper accumulation, comparable to the CASH group, higher amounts of copper can still be excreted. Interestingly, in the N-CASH group, ATP7B is indeed induced compared to healthy controls, emphasizing a possible higher efflux of copper. Furthermore, from the two subclinical disease groups, the N-CASH group is the only one able to recuperate, whereas the CASH group will eventually turn into clinical hepatitis as seen in the DH group (data not shown). Taken together, our data suggest that in the Doberman pinchers copper accumulates in time and, finally, will have its negative effect on copper metabolism and induce oxidative stress.

Oxidative stress has been ascribed to copper toxicosis as one of the most important negative effects [35]. We can confirm this with four different observations: (i) our measurements showed a decrease in mRNA levels of SOD1 and CAT, indicative of a reduction in the enzymatic defence against oxidative stress in all groups with copper accumulation; (ii) a reduction of GSS mRNA levels (glutathione synthesis), indicative for a reduced glutathione level in these groups which is one of the most important non-enzymatic molecules against oxidative stress; (iii) the mRNA levels of GPX1 were significantly increased, indicating an increase in GSH oxidation; (iv) the decrease in GSH was confirmed by measuring total glutathione levels in the DH group towards healthy Doberman pinschers. A similar decrease in expression of anti-oxidant enzymes was observed in ApoE-deficient mice in response to chronic inflammation [36], and inflammatory bowel disease (IBD) [37]. This indicates that chronic inflammation (copper toxicosis, atherosclerosis, IBD) is associated with reduced protection against enhanced exposure to ROS.

Other effects of high copper can also be seen in the measurements on apoptosis and cell-cycle. Measurements on Bcl-2 and XIAP indicate a decrease of protection against apoptosis; however, the most affected hepatocytes will go into necrosis due to the formation of hydroxyl radicals by the Haber-Weiss reaction, which is catalyzed by copper [38]. A striking observation was made measuring p27KIP which was shown to be reduced up to 24-fold in the DH group. This could indicate an induction of cell-cycle compared to healthy controls. This could be ascribed to the renewal of hepatocytes, thus managing the total amount of copper in time.

Whether differential gene expression is cause-or-consequence of hepatitis is unknown. However, it is conceivable that the reduction in copper processing gene products might explain copper accumulation and the subsequent oxidative stress. Furthermore, recent Q-PCR measurements on non-copper related hepatitis and extra hepatic cholestasis suggest that ATP7A and CP are not down-regulated by inflammation or cholestasis (data not shown). Therefore, we can conclude that the decreased expression of these gene products is a Doberman hepatitis specific effect. Other important copper associated gene products such as COX17, ATP7B, and MT1A are probably down-regulated due to inflammation.

## Conclusion

This study is the first to show the effect of prolonged exposure to different copper levels on oxidative stress and copper metabolism in canine livers. Our data supports that: (i) Doberman hepatitis is a new variant of primary copper toxicosis; (ii) there is a clear indication of a reduced copper excretion in the Doberman hepatitis group; (iii) there is a clear correlation between high copper levels and reduced protection against ROS; (iv) this Doberman hepatitis could be a good model to study copper toxicosis and its effects for several human copper storage diseases such as Indian childhood cirrhosis, non-Indian childhood cirrhosis, and idiopathic copper toxicosis, and provide the basis for possible future treatments in dog and even in man.

## Methods

### Dogs

Doberman pinschers were kept privately as companion animals. The dogs were presented to the Department of Clinical Sciences of Companion Animals, Utrecht University, either for a survey investigating the prevalence of Doberman (chronic) hepatitis, as described by Mandigers *et al. *[39] or were referred for spontaneously occurring liver disease. All samples were obtained after written consent of the owner. The procedures were approved by the Ethical Committee, as required under Dutch legislation.

### Groups

Animals were divided in groups based on histopathological examination and quantitative copper analysis. Each group contained both sexes from four to seven years of age. [A possible gender effect was later excluded by looking at the individual data.] Liver tissue of all Doberman pinschers was obtained using the Menghini aspiration technique [40]. Four biopsies, 2–3 cm in length, were taken with a 14-gauge Menghini needle for histopathological examination and quantitative copper analysis and stored for future quantitative PCR and protein investigations. The quantitative copper analysis was performed using instrumental neutron activation analysis via the determination of ^64^Cu [41]. Histopathological biopsies were fixed in 10% neutral buffered formalin, routinely dehydrated and embedded in paraffin. Sections (4 μm thick) were stained with haematoxylin-eosin, van Gieson's stain, reticulin stain (according to Gordon and Sweet), and with rubeanic acid. One experienced board certified veterinary pathologist performed all histological examinations. All diseased groups contained at least six animals that were compared with a group of eight age-matched healthy dogs. Four groups were included in this study (Table 1):

**Table 1:** Doberman pinscher group description

**Group**	**n**	**Hepatic copper**	**Copper concentrations **(mg/kg dry matter)	**Clinical observation**
**Healthy**	8	Normal	100 – 200	No abnormalities
**N-CASH**	6	Normal	< 300	Subclinical hepatitis
**CASH**	6	Elevated copper levels	> 600	Subclinical hepatitis
**DH**	6	Highly elevated copper levels	> 1500	Chronic hepatitis

1) Healthy group (n = 8 dogs), clinically healthy dogs with normal liver enzymes and bile acids. Histopathology of the liver did not reveal histomorphological lesions. Liver copper concentrations were below 200 mg/kg dry matter.

2) Non-copper associated subclinical hepatitis group (N-CASH, n = 6 dogs), dogs with liver enzymes and bile acids within reference values. Although histological examination showed evidence of a slight hepatitis, hepatic copper concentrations were within normal levels, *i.e.*, below 300 mg/kg dry matter. The dogs were classified as suffering from subclinical hepatitis, which most likely was the result of a different etiological factor, such as infections, deficiencies, other toxins, deficient immune status or immune-mediated mechanism [42].

3) Copper associated subclinical hepatitis group (CASH, n = 6 dogs), dogs with liver enzymes and bile acids within reference values. At histopathology these dogs showed centrolobular copper-laden hepatocytes, on occasions apoptotic hepatocytes associated with copper-laden Kupffer cells, lymphocytes, plasma cells and scattered neutrophils. These lesions were classified as subclinical copper-associated hepatitis [43,44]. Hepatic copper concentrations were in all dogs above 600 mg/kg dry matter.

4) Doberman hepatitis group (DH, n = 6 dogs), dogs with chronic hepatitis and elevated hepatic copper concentrations. All dogs were referred with a clinical presentation of hepatic failure (apathy, anorexia, vomiting, jaundice, and in chronic cases sometimes ascites) and died within 2 months after diagnosis from this disease. Heparinized plasma liver enzymes (alkaline phosphatase and alanine aminotransferase) and fasting bile acids were, at least, three times elevated above normal reference values. Abdominal ultrasound revealed small irregular shaped echo dense liver, as performed with a high definition Ultrasound system – HDI 3000 ATL (Philips) – with a 4–7 MHz broad band Faced-array transducer. Histopathology showed chronic hepatitis (Figure 7A) with histological features of fibrosis / micronodular cirrhosis, etc. These lesions are comparable to chronic hepatitis in man [42]. Rubeanic acid staining revealed copper accumulation in hepatocytes and Kupffer cells / macrophages (Figure 7B). Hepatic copper concentrations were in all cases above 1500 mg/kg dry matter.

### RNA isolation and reverse-transcription polymerase chain reaction

Total cellular RNA was isolated from each frozen Doberman liver tissue in duplicate, using Qiagen RNeasy Mini Kit (Qiagen, Leusden, The Netherlands) according to the manufacturer's instructions. The RNA samples were treated with Dnase-I (Qiagen Rnase-free DNase kit). In total 3 μg of RNA was incubated with poly(dT) primers at 42°C for 45 min, in a 60 μl reaction volume, using the Reverse Transcription System from Promega (Promega Benelux, Leiden, The Netherlands).

### Q-PCR of oxidative-stress proteins, copper metabolism and other related signaling molecules

Q-PCR was performed on a total of 17 genes involved in oxidative stress and copper metabolism. Real-time PCR was based on the high affinity double-stranded DNA-binding dye SYBR green I (SYBR^® ^green I, BMA, Rockland, ME) and was performed in triplicate in a spectrofluorometric thermal cycler (iCycler^®^, BioRad, Veenendaal, The Netherlands). For each PCR reaction, 1.67 μl (of the 2× diluted stock) of cDNA was used in a reaction volume of 50 μl containing 1× manufacturer's buffer, 2 mM MgCl_2_, 0.5 × SYBR^® ^green I, 200 μM dNTP's, 20 pmol of both primers, 1.25 units of AmpliTaq Gold (Applied Biosystems, Nieuwerkerk a/d IJssel, the Netherlands), on 96-well iCycler iQ plates (BioRad). Primer pairs, depicted in Table 2, were designed using PrimerSelect software (DNASTAR Inc., Madison, WI). All PCR protocols included a 5-minute polymerase activation step and continued with for 40 cycles (denaturation) at 95°C for 20 sec, annealing for 30 sec, and elongation at 72°C for 30 sec with a final extension for 5 min at 72°C. Annealing temperatures were optimized at various levels ranging from 50°C till 67°C (Table 2). Melt curves (iCycler, BioRad), agarose gel electrophoresis, and standard sequencing procedures were used to examine each sample for purity and specificity (ABI PRISM 3100 Genetic Analyser, Applied Biosystems). Standard curves constructed by plotting the relative starting amount *versus *threshold cycles were generated using serial 4-fold dilutions of pooled cDNA fractions from both healthy and diseased liver tissues. The amplification efficiency, E (%) = (10^(1/-*s*)^-1)·100 (s = slope), of each standard curve was determined and appeared to be > 95 %, and < 105 %, over a wide dynamic range. For each experimental sample the amount of the gene of interest, and of the endogenous references glyceraldehyde-3-phosphate dehydrogenase (GAPDH) and hypoxanthine phosphoribosyl transferase (HPRT) were determined from the appropriate standard curve in autonomous experiments. If relative amounts of GAPDH and HPRT were constant for a sample, data were considered valid and the average amount was included in the study (data not shown). Results were normalized according to the average amount of the endogenous references. The normalized values were divided by the normalized values of the calibrator (healthy group) to generate relative expression levels.

**Table 2:** Nucleotide Sequences of Dog-Specific Primers for Quantitative Real-Time PCR

**Gene**	**Primer**	**Sequence (5'-3')**	**Tm (°C)**	**Product size (bp)**	**Accession number**
GAPDH	Forward	TGT CCC CAC CCC CAA TGT ATC	58	100	AB038240
	Reversed	CTC CGA TGC CTG CTT CAC TAC CTT			
HPRT	Forward	AGC TTG CTG GTG AAA AGG AC	56	100	L77488 /
	Reversed	TTA TAG TCA AGG GCA TAT CC			L77489
SOD1	Forward	TGG TGG TCC ACG AGA AAC GAG ATG	64	99	AF346417
	Reversed	CAA TGA CAC CAC AAG CCA AAC GAC T			
CAT	Forward	TGA GCC CAG CCC TGA CAA AAT G	62	119	AB012918
	Reversed	CTC GAG CCC GGA AAG GAC AGT T			
GSS	Forward	CTG GAG CGG CTG AAG GAC A	62	131	AY572226
	Reversed	AGC TCT GAG ATG CAC TGG ACA			
GPX1	Forward	GCA ACC AGT TCG GGC ATC AG	62	123	AY572225
	Reversed	CGT TCA CCT CGC ACT TCT CAA AA			
CCS	Forward	TGT GGC ATC ATC GCA CGC TCT G	64	96	AY572228
	Reversed	GGG CCG GCC TCG CTC CTC			
p27KIP	Forward	CGG AGG GAC GCC AAA CAG G	60	90	AY455798
	Reversed	GTC CCG GGT CAA CTC TTC GTG			
Bcl-2	Forward	TGG AGA GCG TCA ACC GGG AGA TGT	61	87	AB116145
	Reversed	AGG TGT GCA GAT GCC GGT TCA GGT			
ATOX1	Forward	ACG CGG TCA GTC GGG TGC TC	67	137	AF179715
	Reversed	AAC GGC CTT TCC TGT TTT CTC CAG			
COX17	Forward	ATC ATT GAG AAA GGA GAG GAG CAC	60	127	AY603041
	Reversed	TTC ATT CTT CAA GGA TTA TTC ATT TAC A			
ATP7A	Forward	CTA CTG TCT GAT AAA CGG TCC CTA AA	50	99	AY603040
	Reversed	TGT GGT GTC ATC ATC TTC CCT GTA			
ATP7B	Forward	GGT GGC CAT CGA CGG TGT GC	56	136	AY603039
	Reversed	CGT CTT GCG GTT GTC TCC TGT GAT			
CP	Forward	AAT TCT CCC TTC TGT TTT TGG TT	62	97	AY572227
	Reversed	TTG TTT ACT TTC TCA GGG TGG TTA			
MT1A	Forward	AGC TGC TGT GCC TGA TGT G	64	130	D84397
	Reversed	TAT ACA AAC GGG AAT GTA GAA AAC			
MURR1	Forward	GAC CAA GCT GCT GTC ATT TCC AA	58	122	AY047597
	Reversed	TTG CCG TCA ACT CTC CAA CTC A			
XIAP	Forward	ACT ATG TAT CAC TTG AGG CTC TGG TTT C	54	80	AY603038
	Reversed	AGT CTG GCT TGA TTC ATC TTG TGT ATG			

### Western blot analysis

Pooled liver tissues (n = 6 dogs) were homogenized in RIPA buffer containing 1 % Igepal, 0.6 mM Phenylmethylsulfonyl fluoride, 17 μg/ml aprotinine and 1 mM sodium orthovanadate (Sigma chemical Co., Zwijndrecht, The Netherlands). Protein concentrations were obtained using a Lowry-based assay (DC Protein Assay, BioRad). Thirty five μg of protein of the supernatant was denatured in Leammli-buffer supplemented with Dithiothreitol (Sigma Chemical Co.) for 3 min at 95°C and electrophoresed on 10 % Tris-HCl SDS PAGE polyacrylamide gels (BioRad). Proteins were transferred onto Hybond-C Extra Nitrocellulose membranes (Amersham Biosciences Europe, Roosendaal, The Netherlands) using a Mini Trans-Blot^® ^Cell blot-apparatus (BioRad). The procedure for immunodetection was based on an ECL western blot analysis system, performed according to the manufacturer's instructions (Amersham Biosciences Europe). The membranes were incubated with 4 % ECL blocking solution and 0.1 % Tween 20 (Boom B.V., Meppel, The Netherlands) in TBS for 1 hour under gentle shaking. The incubation of the primary antibody was performed at room temperature for one hour, with a 1:2000 dilution of mouse anti-horse metallothionein (DakoCytomation B.V., Heverlee, Belgium). After washing, the membranes were incubated with horseradish peroxidase-conjugated chicken anti-mouse (Westburg B.V., Leusden, The Netherlands) at room temperature for one hour. Exposures were made with Kodak BioMax Light-1 films (Sigma chemical Co.).

### Total GSH assay

The total amount of GSH was determined by a modified version of a total GSH Determination Colorimetric Microplate Assay according to Allen *et al. *[45], based on the original Tietze macro assay [46]. Protein samples from Doberman hepatitis (n = 6 dogs) and healthy controls (n = 8 dogs) were isolated as described in Western blot analysis and subsequently pooled. Total protein concentration was measured using a Lowry-based assay (DC Protein Assay, BioRad). In short, 50 μl of the cell-lysate (1 mg/ml) was used in triplicate in a 96-wells plate. The lysates were incubated for 5 minutes with 50 μl of 1.3 mM 5,5'dithiobis-2-nitrobenzoic acid (DTNB), and 50 μl GSH reductase (1.5 U/ml). To start the reaction 50 μl of NADPH (0.7 mM) was added to the wells. Absorbance at 450 nm was measured at start and after 5 minutes. The rate of 2-nitro-5-thiobenzoic acid production (yellow product) was measured in delta absorbance per minute and is directly proportionate with the amount of GSH in the samples. A standard curve was added with known concentrations GSH (0 to 20 μM) in order to determine the GSH concentrations in the samples.

### Statistical analysis

A Kolmogorov-Smirnov test was performed to confirm normal distribution of every group, and a Levene's test checked the homogeneity of variances across groups. After both verifications, the statistical significance of the difference between the control group and each particular non-healthy group was determined by using the Student's *t*-Test. The significance level (α) was set at 0.05.

## Authors' contributions

BS performed all Q-PCR measurements and wrote the manuscript. PM participated in its design and coordination and helped to draft the manuscript. BA performed the GSH assays and participated with Western blotting. PB performed the Copper measurements on which our groups are based. TI histochemically examined all samples described in this manuscript. GH performed genotyping on Dobermans and provided theoretical background. JR and LP, conceived of the study, and participated in its design and coordination and helped to draft the manuscript. All authors read and approved the final manuscript.
